# Genomic testing for germline predisposition to hematologic malignancies

**DOI:** 10.1007/s44313-024-00012-y

**Published:** 2024-03-08

**Authors:** Sang Mee Hwang

**Affiliations:** Department of Laboratory Medicine, Seoul National University College of Medicine, Seoul National University Bundang Hospital, Gumiro 173 Beongil-82, Bundanggu, Seongnam, Gyeonggido 13620 South Korea

**Keywords:** Germline predisposition, Hematologic malignancies, Genomic testing, Myeloid neoplasms, Next-generation sequencing

## Abstract

Germline predisposition (GPD) to hematological malignancies has gained interest because of the increased use of genetic testing in this field. Recent studies have suggested that GPD is underrecognized and requires appropriate genomic testing for an accurate diagnosis. Identification of GPD significantly affects patient management and has diverse implications for family members. This review discusses the reasons for testing GPD in hematologic malignancies and explores the considerations necessary for appropriate genomic testing. The aim is to provide insights into how these genetic insights can inform treatment strategies and genetic counseling, ultimately enhancing patient care.

## Introduction

Recent technological advances have allowed high-throughput sequencing to become a routine part of the diagnostic workup for hematologic malignancies, especially myeloid neoplasms (MNs) [1]. Historically, genomic testing of hematological malignancies has focused on identifying somatic alterations within tumor cells. However, the increase of genetic testing has revealed that certain hematologic malignancies can be attributed to either inherited or de novo germline mutations [2]. Bone marrow failure syndromes, such as Fanconi anemia (FA) and Diamond-Blackan anemia (DBA), are well-known diseases with germline predisposition (GPD) to hematologic malignancies, often exhibiting non-hematological findings and presenting in childhood [3]. However, expanding knowledge has led to the discovery of MNs with GPD that arise without preexisting hematological abnormalities or are diagnosed later in life.

The revised 4th Edition of the World Health Organization (WHO) classification of tumors includes MN with GPD, and the latest 5th Edition of the WHO expands this category to incorporate additional genes [4]. MNs with GPD are classified into three groups: 1) without a preexisting platelet disorder or organ dysfunction, 2) with a preexisting platelet disorder, and 3) with potential organ dysfunction, including RASopathies, Down syndrome, bone marrow failure syndrome, and telomere biology disorders (Table 1). The International Consensus Classification (ICC) proposes a similar classification termed “hematological neoplasms” with GPD instead of MNs and includes an additional category of acute lymphoblastic leukemia with GPD containing a germline *PAX5*, *IKZF1* mutation [5]. Although germline pathogenic/likely pathogenic (P/LP) variants mostly lead to MNs, lymphoid malignancies, including acute lymphoblastic leukemia and lymphomas, have also been identified.

**Table 1:** WHO 5th Edition and ICC for myeloid/hematologic neoplasms with GPD

WHO 5th Edition	ICC
Myeloid neoplasms with GPD *without a pre-existing platelet disorder or organ dysfunction*	Hematologic neoplasms with GPD *without a constitutional disorder affecting multiple organ systems*
Germline *CEBPA* P/LP variant (*CEBPA*-associated familial AML)	Myeloid neoplasms with germline *CEBPA* mutation
Germline *DDX41* P/LP variant^a^	Myeloid or lymphoid neoplasms with germline *DDX41* mutation
Germline *TP53* P/LP variant (Li Fraumeni syndrome)^a^	Myeloid or lymphoid neoplasms with germline *TP53* mutation
Myeloid neoplasms with GPD *and pre-existing platelet disorder*	Hematologic neoplasms with GPD *associated with a constitutional platelet disorder*
Germline *RUNX1* P/LP variant^a^ (Familial platelet disorder with associated myeloid malignancy, FPD-MM)	Myeloid or lymphoid neoplasms with germline *RUNX1* mutation
Germline *ANKRD26* P/LP variant^a^ (Thrombocytopenia 2)	Myeloid neoplasms with germline *ANKRD26* mutation
Germline *ETV6* P/LP variant^a^ (Thrombocytopenia 5)	Myeloid or lymphoid neoplasms with germline *ETV6* mutation
Myeloid neoplasms with GPD *and potential organ dysfunction*	Hematologic neoplasms with GPD *associated with a constitutional disorder affecting multiple organ systems*
Germline *GATA2* P/LP variant (GATA2 deficiency)	Myeloid neoplasm with germline *GATA2* mutation
Germline *SAMD9* P/LP variant (MIRAGE syndrome)	Myeloid neoplasm with germline *SAMD9* mutation
Germline *SAMD9L* P/LP variant (SAMD9L-related ataxia pancytopenia Syndrome)	Myeloid neoplasm with germline *SAMD9L* mutation
RASopathies (Neurofibromatosis type 1, CBL syndrome, Noonan syndrome or Noonan-syndrome-like disorders^a^)	Juvenile myelomonocytic leukemia associated with neurofibromatosis
	Juvenile myelomonocytic leukemia associated with Noonan-syndrome-like disorder (CBL syndrome)
Down syndrome^a^	Myeloid or lymphoid neoplasms associated with Down syndrome
Bi-allelic germline *BLM* P/LP variant (Bloom syndrome)	
Bone marrow failure syndromes:	Myeloid neoplasms associated with bone marrow failure syndromes:
- FA	- FA
- SDS	- SDS
- DBA	- DBA
- Severe congenital neutropenia	- Severe congenital neutropenia
- Telomere biology disorders	- Telomere biology disorders including dyskeratosis congenita
	Acute lymphoblastic leukemia with GPD^b^
	Acute lymphoblastic leukemia with germline *PAX5* mutation
	Acute lymphoblastic leukemia with germline *IKZF1* mutation

*Abbreviations*: *P* Pathogenic, *LP* Likely pathogenic

^a^Lymphoid neoplasms can also occur

^b^Down syndrome and germline mutations in *ETV6* or *TP53* also predispose to acute lymphoblastic leukemia

This review focuses primarily on the genomic testing for MNs with GPD and discusses points of consideration for the choice of specimen, type of testing, and interpretation.

### Why test for GPD in hematologic malignancies?

Recognizing GPD can guide therapeutic decisions, appropriate genetic counseling, familial screening, and surveillance [6, 7]. However, patients with MNs with GPD may not have a family history of cancer, may lack a clinical phenotype, or have syndromic features that may be mild or unrecognized. In such instances, without testing for germline variants, the underlying GPD may not be identified. A study on myelodysplastic syndrome (MDS) showed that GPD occurred in patients of any age, even in those aged > 70 years [8]. This study showed that nearly 7% of the patients with MDS carried P/LP variants. GPD for bone marrow failure, DNA repair, and telomere biology disorders occurred at ages of < 40 years, whereas checkpoint disorders or germline variants in *DDX41* occurred at a later age. A study of 1120 patients with pediatric cancer showed that 8.5% had germline mutations in cancer-predisposing genes and only 40% had a family history of cancer [9]. Thus, the 2022 European LeukemiaNet recommendation for the diagnosis and management of acute myeloid leukemia (AML) states that GPD should be considered in patients with any hematological malignancy, irrespective of age [10].

GPD with underlying bone marrow failure syndrome may require therapeutic modifications. In cases of GPD such as FA, hematopoietic stem cell transplantation (HSCT) with reduced-intensity conditioning is required because of the inherent hypersensitivity of these patients to genotoxic therapies. Significant treatment-related toxicities and prolonged cytopenia can occur with standard regimens [11, 12]. In a cohort of patients diagnosed with severe aplastic anemia who underwent HSCT, P/LP germline variants in 42 genes associated with inherited bone marrow failure syndromes were identified in 16.5% (121/732) of the patients [13]. In patients with *GATA2* deficiency, various infectious complications during therapy should be considered [14]. Donor selection for HSCT is important as germline variants may be present in family members. Donor-derived malignancies have been reported in MNs with GPD for *CEBPA* [15], *DDX41* [16], and *GATA2* [17], and stem cell donors carrying pathogenic germline variants have demonstrated inferior outcomes characterized by challenges in stem cell mobilization or delayed engraftment failures [18].

Universal screening of individuals for GPD of MN is not currently the standard of care, but the American Society for Clinical Oncology recommends screening for hereditary cancer syndromes when (1) there is a personal or familial history suggestive of a hereditary cancer syndrome, (2) the screening test results can be accurately interpreted, and (3) the outcomes of the screening will contribute to the diagnosis or assist in managing the patient or family members at risk [19]. The Nordic guidelines recommend GPD testing when hematologic malignancies are diagnosed in a patient with a family history or signs/symptoms indicative of a hereditary condition, when gene variants are suspected to be germline based on somatic testing, or when MDS/AML is diagnosed in patients aged < 50 years in the presence of chromosome 7 aberrations [7].

### How to test for GPD to hematologic malignancies

The challenge in diagnosing germline variants in hematologic malignancies lies in the fact that peripheral blood is not an ideal source for genetic testing because the hematopoietic cells themselves are the source of the tumor. The recommended specimen for confirming germline variants is non-hematopoietic, such as skin fibroblasts, thus enabling less contamination by blood cells. However, this requires an additional procedure for a skin biopsy and additional time for culturing [20]. Other specimens, such as nail clippings, hair bulbs, buccal swabs, bone marrow, or peripheral blood at remission status, may be used but with caution. For saliva and buccal swabs, contamination by white blood cell may complicate interpretation, and a sufficient amount of DNA should be obtained from nail clippings and hair bulbs [21, 22]. Somatic mosaicism may occur, leading to situations in which a germline variant is not identified due to somatic reversion in blood cells; this has been described in cases involving *RUNX1*, *SAMD9*, and *SAMD9L* [23]. Inappropriate specimens can lead to false-negative or false-positive results, which may have a critical impact. Thus, the use of skin fibroblast samples are recommended for testing.

### Selection of genetic testing methods

When an appropriate sample has been selected, a methodology to identify GPD variants should be considered. The selection depends on regulatory aspects, costs, and availability within the institution. Therefore, targeted gene panels using next-generation sequencing (NGS) should be considered. The genes included in the panels are most likely based on the current classifications of GPD, and the gene lists for hereditary disorders can be reviewed through the Gene Curation Coalition (GenCC) [24]. Importantly, differences in hotspots for somatic and germline variants may exist in certain genes. These differences should be considered during the panel selection, testing, and interpretation. In the United Kingdom, the National Health Service (NHS) in England sanctioned the implementation of whole-genome sequencing (WGS) as a standard care practice for all patients with acute leukemia. This approach involves performing paired tumor and germline WGS, which facilitates the identification of a greater number of germline variants than existing methods [25]. Germline testing strategies vary based on care plans and institutions in the United States and Korea. The availability of germline panels is increasing; however, WGS is not yet in clinical use in most scenarios.

For familial AML with *CEBPA* mutations, N-terminal germline variants with acquired somatic mutations in the C-terminus have mostly been reported. The presence of multiple *CEBPA* mutations or truncating alleles in tumor-based molecular profiling may warrant additional germline testing [26]. In myeloid neoplasms with *DDX41* variants, 85% are germline and > 95% of the truncating alleles are germline variants. Among *DDX41* variants, the c.3G > A, p.M1? start-loss allele is often detected as a germline variant. However, a low variant allele frequency (VAF) may be observed in tumor profiling because of technical issues, which can lead to misinterpretation as a somatic variant, necessitating caution in interpretation [27]. Different ethnic groups have different variant frequencies: Japanese and Korean individuals are enriched with c.1496dup, whereas c.3G > A and c.415_418dup are more common in individuals of Northern European descent [28–30]. The presence of multiple *DDX41* variants, especially those with high VAF, suggests a germline mutation.

In the case of *RUNX1*, the same variants have been identified in both somatic and germline settings within hematologic malignancies, highlighting the challenge of determining when to use germline confirmation. Germline *RUNX1* variants are distributed throughout the gene, necessitating sequencing the entire gene. These variants include missense, nonsense, frameshift, and whole-exon deletions or duplications [31]. A previous study involving 45 families revealed that partial or whole deletions of the *RUNX1* locus comprised a significant portion, along with splice sites and intragenic duplications [32]. Germline *ANKRD26* variants are located in the 5’ untranslated regions (UTR) of c.-116 to c.-134, leading to overexpression of ANKRD26 owing to the failure of regulation by transcription factors RUNX1 and FLI1; thus, this region should be included in analyses [33, 34]. Germline *ETV6* mutations are typically found in the N-terminal central regulatory domain and C-terminal ETS motif. Notably, the distribution of somatic *ETV*6 variants showed a domain pattern similar to that of the germline variants.

For GATA2 deficiency, germline mutation involves truncating mutations, missense mutations within zinc finger 2, and noncoding variants in the + 9.5-kb regulatory region of *GATA2.* Most adolescent individuals with monosomy 7 MDS carry an underlying GATA2 deficiency [35]; thus, genetic testing for *GATA2* may be necessary and is recommended in the Nordic guidelines [7]. Germline variants of *SAMD9/SAMD9L* can be accompanied by acquired loss-of-function mutations in the same gene (in cis) or by monosomy 7. This leads to loss of the mutated germline allele, complicating genetic testing because only the wild-type *SAMD9/SAMD9L* allele remains [36, 37].

As somatic tumor panels are widely used in hematologic malignancies, suspicious germline variants may be encountered in these panels. Additional testing is necessary to confirm that the variants are germline variants [38]. Drazer et al. identified germline variants in 21% of patients using tumor sequencing panels for hematologic malignancies and showed that a VAF of > 0.4 in the gene of interest may be predictive of a germline origin [39]. Experts have suggested that *RUNX1* and *ETV6* should be carefully assessed for germline origin if detected with a high VAF. It has been suggested that certain mutation patterns may indicate germline origin. These include the presence of mutations in both alleles of a gene, gene mutations accompanied by copy number variations (CNVs) in the same gene, large exon-spanning duplications or deletions, and the persistence of gene mutations in follow-up studies, even during remission [40].

There are numerous associated genes in MNs with GPD in bone marrow failure syndromes, such as FA, DBA, and Schwachman-Diamond syndrome (SDS). In FA, over 20 genes are implicated and for some genes like *FANCA*, 40% of the variants are detected through deletion/duplication analysis, necessitating careful consideration of the testing type [41]. Biallelic pathogenic variants of *SBDS* have been identified in most patients with SDS, with mutations commonly occurring within exon 2, c.258 + 2 T > C and c.183_184delinsCT [42]. Challenges in genetic testing include the presence of an *SBDSP1* pseudogene, which shares 97% sequence identity with *SBDS*, complicating the identification or estimation of VAF because reads may be misaligned to *SBDSP1* [43]. It is crucial to determine whether the variants are cis or trans, as a single heterozygous *SBDS* mutation with one wild-type allele is insufficient for the disease. Variants in other genes such as *EFL1* and *SRP54* may result in clinical features that overlap with those of SDS. The DBA genotype is heterogeneous and involves more than 20 ribosomal protein genes, with *RPS19* being the most frequently mutated. Large deletions were found in *RPS17*, *RPL35a*, and *RPS19*, which should be considered during testing.

### Interpretation of germline variants

The process of interpreting germline variants adheres to the guidelines established by the American College of Medical Genetics and Genomics (ACMG) and the Association for Molecular Pathology (AMP) [44], which differs from the interpretation of somatic variants based on the AMP/College of American Pathologists/ACMG guideline [45]. Germline variants are classified based on a five-tier system: pathogenic, likely pathogenic, benign, likely benign, and uncertain significance. The rules for combining criteria to classify sequence variants are complex and require the assessment of evidence of pathogenicity (very strong, strong, moderate, and supporting) or benign (stand-alone, strong, and supporting). However, as the guideline is a general rule and gene-specific guidelines are lacking for the majority of genes included for testing the GPD of hematologic malignancies, practical recommendations have been proposed for interpreting germline variants for hematologic malignancies, bone marrow failure, and chronic cytopenia [46]. This guide provides thresholds for minor allele frequencies, in silico predictions, the use of constraint Z-scores, and functional evidence required for assessing pathogenicity. For the same variant, differences in variant interpretation may exist between the somatic and germline settings because different variant interpretation guidelines are used for different purposes and needs. Mutation type, minor allele frequency, published studies, in silico tools, and germline databases can be considered for both somatic and germline testing. However, for somatic variants, Food and Drug Administration (FDA)-approved therapies, somatic databases, variant frequencies in tumors, and professional guidelines should be considered when segregation information, in trans findings, and patient phenotype are also taken into account. Clinicians should be aware of the difference [47, 48]. Specific guidelines for variant interpretation have been proposed for only a few genes or conditions, such as *RUNX1* [49, 50], *TP53* [51], and RASopathy [52]; however, the list is expanding and ClinGen Expert panel reports should be prioritized. One report showed that only 21% of *RUNX1* variants listed in ClinVar are clinically significant, thus suggesting caution in variant interpretation [49].

The ACMG has proposed points for consideration when reporting germline variations in patients undergoing tumor testing [38]. Individuals undergoing tumor testing must provide informed consent, acknowledging the potential discovery of germline pathogenic variants. It should be clearly communicated to patients that the identification of such variants will lead to a referral for genetic consultation and may necessitate confirmatory germline testing. Additionally, confirmatory germline testing should be conducted in a clinical laboratory that specializes in this area, with the results communicated by both qualified and experienced clinicians.

### Genetic counseling and surveillance

Most guidelines recommend that genetic testing be performed with pre- and post-test counseling [7, 19]. Currently, no standard surveillance guidelines are available for unaffected carriers of germline P/LP variants. However, peripheral blood cell counts, family history, and physical examinations are performed with an initial bone marrow workup and consultation for HSCT [7, 53]. Surveillance in asymptomatic carriers depends on the age and specific disorder, as some variants are prone to result in MNs at a young age, whereas others, such as *DDX41*, manifest later in life. Expert panels have recommended surveillance for children with leukemia-predisposing conditions because some cases may benefit from preemptive treatment with allogeneic HSCT [54]. Early referral to a transplant specialist and donor identification should be considered.

## Conclusions

GPD is frequently observed in patients with myeloid or hematological neoplasms. Importantly, 5–10% of myeloid neoplasms have an underlying GPD and appropriate testing should be performed regarding the sample type, testing methodologies, and interpretation. Clinicians and pathologists must continually update their knowledge regarding hematologic malignancies with GPD to ensure that patients receive the most informed and effective management.

## Data Availability

No datasets were generated or analysed during the current study.
